# PET/MR imaging: current status and future direction

**DOI:** 10.1186/1470-7330-15-S1-O32

**Published:** 2015-10-02

**Authors:** Heinz-Peter Schlemmer

**Affiliations:** 1 German Cancer Research Center, Department of Radiology, INF 280, 69120 Heidelberg, Germany

## 

PET/CT has emerged as an important tool in oncology to detect, characterize, and follow-up various tumor entities. The integrated technology includes the respective advantageousness of PET and CT in a synergistic manner combining high sensitivity of PET with transmission correction and anatomic referencing by CT. PET/CT has been proven to be fast, robust and meanwhile widely available in oncologic centers. But for several tumor entities, e.g. cancers of the brain, liver, cervix or prostate, MRI would be beneficial compared to CT due to the superior soft tissue contrast and additionally functional information about e.g. tumor perfusion (DCE, T2*) and diffusion (DWI). On the other hand, sequential MR and PET/CT imaging has several limitations: patient repositioning may result in spatial mismatch and the various ways of the correction of misalignment are time consuming and limited; it is furthermore uncomfortable for critically ill patients, costly in terms of time and personal resources and inefficient from a work-flow perspective. Scientific and clinical considerations therefore argue for the benefits of simultaneous coregistration of MR and PET imaging in oncology. Simultaneous PET/MR furthermore reduces the X-ray exposures of the patients, which is an important issue particularly for young patients and patients who need multiple follow-up examinations.

Sequential and fully integrated whole-body 3.0 Tesla PET/MR scanners are meanwhile available from Philips, Siemens and GE. Initial limitations of the simultaneous PET/MR technology concerning the attenuation correction with MR alone have been extensively addressed. PET image reconstruction with accurate attenuation correction as well as quantification of local radiotracer uptake, i.e. calculation of the SUV, is now possible using the MR data [1]. Furthermore, simultaneous temporal and spatial coregistration of PET and MR data has also been successfully applied for MRI-gated PET measurements of moving organs/tumors in e.g. bronchial or liver cancer [2]. Initial feasibility studies have proven that PET/MR imaging can be advantages for tumor detection, staging as well as for therapy monitoring and follow-up in e.g. lung cancer, head and neck cancers, breast cancer, liver cancer, prostate cancer, lymphoma, seminal carcinoma etc. [3-7]. Beneficial applications are in particular for comprehensive management of solid tumors in children concerning tumor detection, characterization as well as treatment monitoring [8]. Promising applications have been published in particular for prostate cancer concerning whole-body PET/MR with ^68^Ga-labelled prostate-specific membrane antigen (PSMA) ligand [9].

But despite an increasing body of knowledge about the application of sequential and simultaneous PET/MR in oncology various open questions still exist concerning the scientific and medical prospects and limitations. What unmet radiological needs are addressed by PET/MR from a scientific and clinical point of view? Is a hardware integration of PET and MR actually required from a clinical point of view? What are the clinical indications in which simultaneous PET/MR achieves higher diagnostic accuracy compared to subsequent MR and PET/CT imaging? Could sequential PET/CT and MR imaging followed by software-based image coregistration be sufficient in particular clinical situations? What are the essential added values of PET/MR compared to PET/CT? Can PET/MR be economically reasonable and reduce overall costs for therapy planning monitoring and follow-up?
